# Silica Perturbs Primary Cilia and Causes Myofibroblast Differentiation during Silicosis by Reduction of the KIF3A-Repressor GLI3 Complex

**DOI:** 10.7150/thno.37049

**Published:** 2020-01-01

**Authors:** Shifeng Li, Zhongqiu Wei, Gengxu Li, Qiaodan Zhang, Siyu Niu, Dingjie Xu, Na Mao, Si Chen, Xuemin Gao, Wenchen Cai, Ying Zhu, Guizhen Zhang, Dan Li, Xue Yi, Fang Yang, Hong Xu

**Affiliations:** 1 Medical Research Center, Hebei Key Laboratory for Organ Fibrosis Research, North China University of Science and Technology, Tangshan, China; 2 Basic Medicine College, North China University of Science and Technology, Tangshan, China; 3 College of Traditional Chinese Medicine, North China University of Science and Technology, Tangshan, China; 4 School of Public Health, North China University of Science and Technology, Tangshan, China; 5 Department of Neurosurgery, Tangshan People's Hospital, Tangshan, China; 6 Basic Medical College, Xiamen Medical Collage, Xiamen, China

**Keywords:** Primary cilia, Kinesin family member 3A, Glioma-associated oncogene homolog 3, silicosis, fibroblast

## Abstract

The purpose of this study was to determine the effects of Kinesin family member 3A (KIF3A) on primary cilia and myofibroblast differentiation during silicosis by regulating Sonic hedgehog (SHH) signalling.

**Methods**: Changes in primary cilia during silicosis and myofibroblast differentiation were detected in silicotic patients, experimental silicotic rats, and a myofibroblast differentiation model induced by SiO_2_. We also explored the mechanisms underlying KIF3A regulation of Glioma-associated oncogene homologs (GLIs) involved in myofibroblast differentiation.

**Results**: Primary cilia (marked by ARL13B and Ac-α-Tub) and ciliary-related proteins (IFT 88 and KIF3A) were increased initially and then decreased as silicosis progressed. Loss and shedding of primary cilia were also found during silicosis. Treatment of MRC-5 fibroblasts with silica and then transfection of *KIF3A*-siRNA blocked activation of SHH signalling, but increased GLI2^FL^ as a transcriptional activator of *SRF*, and reduced the inhibitory effect of GLI3^R^ on *ACTA2*.

**Conclusion**: Our findings indicate that primary cilia are markedly altered during silicosis and the loss of KIF3A may promote myofibroblast differentiation induced by SiO_2_.

## Introduction

Silicosis characterised by the formation of silicotic nodules is the most serious occupational disease in China and caused by long-term inhalation of dust 1, 2. According to our previous study on downregulation of acetylated tubulin α (Ac-α-Tub) in silicotic rats 3, we speculated that primary cilia, which are microtubule-based cellular protrusions, may also exhibit some changes during the progression of silicosis.

The primary cilium is a solitary, non-motile organelle that acts as a mechanosensory and chemosensory antenna protruding from the plasma membrane of many mammalian cell types 4. The characteristics of many acquired diseases include abnormalities in both the cilium structure and function. Excessive ciliogenesis is found in the hyperplastic alveolar epithelium and fibroblastic foci of idiopathic pulmonary fibrosis (IPF) 5 as well as cardiac fibrosis 6. Moreover, primary cilium loss contributes to a switch from reversible to irreversible kidney injury in unilateral ureteral occlusions (UUOs) 7. Detection of the primary cilium length, primary cilium fragments, or ciliary proteins in urine can be useful to evaluate the severity of kidney diseases, at least in acute kidney injury (AKI) 8. Disruption of cilia can induce epithelial-myofibroblast transition (EMyT) 9. Emerging observations implicate dysfunctional primary cilia in fibrosis of many tissues and organs, and primary cilia appear to be necessary to initiate the transition and sustained activation of myofibroblasts 10.

Kinesin family member 3A (KIF3A) is a subunit of heterotrimeric kinesin-2 and acts as a microtubule plus end-directed motor protein that regulates the cilium length to maintain primary cilia for compensation upon alteration 11. The *Kif3a* knockout mouse is an established non-orthologous mouse model of cystic kidney disease characterised by loss of primary cilia, rapid cyst growth, and fibrosis 12. Moreover, tissue-specific loss of *Kif3a* in pancreatic cells results in severe pancreatic fibrosis 13. However, the mechanisms underlying the influence of *Kif3a* knockout on fibrosis are still unclear.

Recently, we reported a major role of the Sonic hedgehog (SHH) pathway in silicosis 14. SHH relieves the inhibitory effects of patched-1 (PTC1) on smoothened proteins (SMO), both of which are ciliary membrane-bound receptors, and initiates the signalling cascade via the Glioma-associated oncogene homolog (GLI) family (GLI1-3) of transcription factors 15. GLI1 is a transcriptional activator and *GLI1* is a target gene of SHH signalling. Therefore, its function is reinforced by a positive feedback loop through SHH pathway activation. GLI2 and GLI3 have both full-length forms (GLI^FL^) as transcriptional activators and proteolytically processed forms as transcriptional repressors (GLI^R^). GLI3^R^ performs its function exclusively as a strong repressor of SHH pathway target genes 16, 17. GLI proteins interact selectively and synergistically with KIF3A 18. In addition, *kif3a*^-/-^ mouse embryonic fibroblasts (MEFs) express a significantly lower level of GLI3^R^ 19.

In the present study, we found loss of primary cilia and shedding of ciliary proteins accompanied by silicosis progression, indicating that silica disrupts the structure and function of primary cilia in fibroblasts. We also found that silica activated SHH signalling to promote myofibroblast differentiation, and that initial primary cilium enlargement was followed by complete deciliation. Knockdown of *KIF3A* inhibited SHH signalling activation, but also increased CLI2^FL^ and decreased GLI3^R^ transcription to promote myofibroblast differentiation. Our findings indicate that primary cilia are markedly altered during silicosis and loss of KIF3A may promote myofibroblast differentiation.

## Methods

### Rat model

Animal studies were conducted with the protocol approved by the Institutional Animal Care and Use Committee of the North China University of Science and Technology, Tangshan, China (2013-038). Three-week-old male Sprague-Dawley (SD) rats with 180±10 g of body weight were purchased from Vital River Laboratory Animal Technology Co. Ltd. (SCXY 2009-0004, Beijing, China). They were fed and housed in the SPF-class laboratory at North China University of Science and Technology in accordance with the National Institutes of Health (NIH) guidelines.

The silicotic model was made using a HOPE MED 8050 exposure control apparatus (HOPE Industry and Trade Co. Ltd, Tianjin, China) as previous report 20. Briefly, the rats were exposed to SiO_2_ (99% particle size of 0.5-10 μm, S5631, Sigma-Aldrich, St. Louis, MO, USA) for 3 h daily for 4, 12, and 24 weeks (n=10). The SiO_2_ concentration is 50±10 µg/m^3^. The rats in the corresponding control group were exposed to the same condition of pure air without SiO_2_ for 3 h daily for 4, 12, and 24 weeks (n=10).

### Human lung tissue specimens and Bronchoalveolar lavage fluid (BALF)

The human study was approved by the Medical Ethics Committee of North China University of Science and Technology (2015-046). Written informed consent was obtained from each subject to confirm their voluntary participation in this study.

Slides of formalin-fixed, paraffin-embedded lung tissue sections of coal worker's pneumoconiosis (CWP) were obtained from the Department of Pathology of North China University of Science and Technology, Tangshan, China. The autopsies of silica-exposed workers were from 32 patients with an average of 19 years of occupational exposure history who were diagnosed with silicosis using the diagnostic criteria for occupational pneumoconiosis of China (GBZ 70-2015) and evaluated by occupational pulmonary pathologists. The pericarcinomatous tissue served as the negative control. The typical pathological changes in silicotic patients, including macrophage alveolitis, cellular silicotic nodules, and fibrous silicotic nodules, are shown in Figure S1.

BALF samples were collected from patients who underwent massive whole lung lavage (instillation and recovery of 0.9% sterile saline in the bronchopulmonary segment) without clinical contraindications from China's Coal Miners Beidaihe Sanatorium. The study included 48 patients who were diagnosed with silicosis by the Occupational Diseases Committee, and those patients were divided into three grades, i.e. silicosis phase I (16 cases), II (16) and III (16), by diagnostic criteria for occupational pneumoconiosis of China (GBZ 70-2015). Stage 0+ workers (8 cases) were chosen as the control group. All of the subjects had no serious diseases in the heart, brain, liver, or kidneys, and those over 65 years of age were excluded. All of the participants were male. The recovered fluid was condensed via centrifuge at 600×g at room temperature (RT) for 15 min, and the supernatants were collected for detection. The demographic characteristics, occupational exposure, and pulmonary function tests of the enrolled subjects who underwent massive whole lung lavage are summarised in Table 1.

### Cell culture and treatments

A human embryo lung fibroblast MRC-5 cell line was purchased from the Chinese Academy of Sciences Cell Library (TCHu150, Shanghai, China). The MRC-5 fibroblasts were treated with SiO_2_ at a concentration of 50 μg/cm^2^. Culture medium was used as a vehicle control.

The cells were treated with 100 nmol/L of SMO agonist (SAG, CSN19210, CSNpharm, Chicago, IL, USA) for activating SHH signalling, 4 μg/L of specific GLI antagonist 61 (GANT61) (S8075, Selleckchem.com inhibitor expert, Munich, Germany) for inhibiting GLI1/2, and 20 μmol/L of GDC-0449 (S1082, Selleck, China) for inhibiting SMO 21. The degradation of GLI3^R^ was examined by 50 mg/L of cycloheximide (CHX, 239763-M, Sigma-Aldrich St. Louis, MO, USA). Media was used with a final FBS concentration of 0.5%.

### RNA interference

The MRC-5 fibroblasts were cultured in antibiotic-free medium and treated with lipofectamine 2000 reagent (11668, Invitrogen, Carlsbad, CA, USA) and a siRNA mixture for 8 h, followed by exchange medium, and maintained for 48 h.

The sequences targeting human *KIF3A* were as follows: 1) Sense: 5′ CUGCGUCAGUCUUUGAUGA dTdT 3′; Antisense: 5′ UCAUCAAAGACUGACGCAG dTdT 3′; 2) Sense: 5′ CUUCGACUUCAGAUGCUUA dTdT 3′; Antisense: 5′ UAAGCAUCUGAAGUCGAAG dTdT 3′; 3) Sense: 5′ AGGCUAGAGCUGAAUUAGA dTdT 3′; Antisense: 5′ UCUAAUUCAGCUCUAGCCU dTdT 3′ (RuiBo Biological Technology Co., Ltd, Guangzhou, China).

### Plasmid transfection

Vectors encoding homo sapiens kinesin family member 3A (KIF3A) (NM_001300791) cDNA clone (TrueClone cDNA) were purchased from OriGene Technologies, Inc. Empty vector *NC-cpDNA* and *KIF3A-cpDNA* were transfected using Lipofectamine 2000 reagent (11668, Invitrogen, Carlsbad, CA, USA), according to the manufacturer's recommendations.

### Histopathological examination

Lung tissues were fixed, embedded, and transversed. Slices (4 μm) were stained with haematoxylin and eosin (Sigma-Aldrich, St. Louis, MO, USA), Sirius Red (Maxin Company, Fuzhou, China), and Masson's trichrome staining (Maxin Company, Fuzhou, China). Images were obtained using an Olympus DP80 light microscope (Olympus Corporation, Tokyo, Japan). The histopathological images were only for pathological observation, and the pathological figures were reviewed by two clinical pathologists.

### Immunofluorescence staining (IF)

IF staining was performed as previously reported 20. Paraffin sections of the lung tissue and cell slides were incubated with α-SMA (1184-1, Eptomics, Burlingame, CA, USA, 1:300), Ac-α-Tub (sc-23950, Santa Cruz Biotechnology, Santa Cruz, CA, USA, 1:200), γ-Tubulin (ab210797, Abcam, Cambridge, MA, USA, 1:200) and ADP-ribosylation factor-like protein 13B (ARL13B) (GTX122703, GeneTex, Inc., Irvine, CA, USA, 1:200). The slides were then counterstained with DAPI (5 g/L, Beyotime, Haimen, China) and were visualised using an Olympus DP80 microscope (Olympus Soft Imaging).

### Immunohistochemistry staining (IHC)

IHC staining was performed as previously described 20 and with primary antibodies against α-SMA (1184-1, Eptomics, Burlingame, CA, USA, 1:300) and Ac-α-Tub (ab125356, Abcam, Cambridge, MA, USA, 1:300). Immunoreactivity was visualised with DAB (ZLI-9018, ZSGB-BIO, Beijing, China). Brown staining was considered a positive result.

### Western blotting analysis

Western blot was performed as previously described 20. Primary antibody used in our studies included Ac-α-Tub, collagen type I (COL I), KIF3A (ab125356, ab34710, ab11259, Abcam), intraflagellar transport protein (IFT) 88 (MFG42775, Aviva Systems Biology), ARL13B (GTX122703, GeneTex), α-SMA, (1184-1, Eptomics), MRTF-A, SHH, SMO, GLI1 (A12598, A7726, A3274, and A8387, ABclonal, Wuhan, China), SRF, PTC1, α-Tub (AF6160, AF5202, AF7010, Affinity Biosciences, Cincinnati, OH, USA), Ac-α-Tub, LaminB, GLI2, GLI3, and GAPDH (sc-23950, sc-56143, sc-271786, sc-74478, and sc-25778, Santa Cruz Biotechnology, Santa Cruz, CA, USA).The results were normalised with loading control and expressed as the fold of the specific bands to the control group. Western blotting was repeated at least three times.

### Co-immunoprecipitation (CoIP)

The interaction of KIF3A with GLI2 and GLI3 was evaluated by CoIP. The cells were lysed in RIPA (R0020, Solarbio Life Science, Beijing, China) buffer containing 1% protease inhibitors. Then 30 µl of sepharose beads (FO115, Santa Cruz Biotechnology, Santa Cruz, CA, USA) and cell lysates (2 g/L) were mixed to a volume of 400 µl and incubated for 2 h at 4 °C on a shaker for preclearing. The clear supernatant was incubated overnight with anti-KIF3A, anti-IgG antibody, and Protein A sepharose overnight at 4 °C. The beads were collected and washed 3 times with PBS before being boiled in 2× loading buffer at 95 °C for 5 min. Western blotting was used to analyse the CoIP results.

### Chromatin immunoprecipitation (ChIP) assay

ChIP assays were performed following the indicated protocol of a Chromatin Immunoprecipitation Kit (17-371, EMD Millipore, Temecula, CA, USA). 1×10^7^ cells were used for each ChIP enrichment. Briefly, MRC-5 fibroblasts were stimulated by SiO_2_ for 12 h and treated with or without *KIF3A*-siRNA for another 24 h. The cells were then cross-linked with formaldehyde (with a final concentration of 1%) for 10 min at RT and then stopped by glycine (with a final concentration of 1.25 mol/L) for 5 min. Shearing cross-linked DNA to 200-1000 base pairs in length was performed by sonication. The sonicated chromatin was incubated with 2 μg of anti-GLI2 (DF7541, Affinity Biosciences, Cincinnati, OH, USA), positive control, and normal mouse IgG overnight with rotation at 4 °C, followed by elution of the protein/DNA complexes, reverse cross-linking, and DNA purification. ChIP-qPCR was performed and the results were calculated as a percentage of the input DNA. The qPCR reagent assembly for 1 reaction was as follows: ddH_2_O (9.5 ul, Corning, USA), Primer mix (1ul, Invitrogen), SYBR Green MasterMix (12.5 ul, Takara, Shiga, Japan). After initial denaturation for 10 min at 4 °C, they underwent denaturing 50 times at 94 °C for 20 s and annealing and extension at 60 °C for 1 min. The relative gene expression was calculated using the 2 -ΔΔCT method.

The sequences of the primers used for qPCR are listed as follows: hα-SMA, F: 5′-ACTGAGCGTGGCTATTCCTCCGTT-3′, R: 5′-GCAGTGGCCATCTCATTTTCA-3′. hSRF, F: 5′-ACTGAGCGGCTATTCCTCCGTT-3′, R: 5′-GCAGTGGCCATCTTTTTCA-3′.

### Statistical analyses

SPSS 22.0 software was used to perform the statistical analysis. All of the results were presented as median±standard deviation (SD). Analysis of variance (ANOVA) was performed to compare the outcomes of the averages of more than two groups, constant variance using the LSD test, and heterogeneity of variance using Tamhane's test. The independent-samples *t*-test was used to compare the means of the two samples. The chi-squared test was used to compare the qualitative data obtained in the analysed groups, *P*-values <0.05 were considered statistically significant. The correlations were analysed between the changes in KIF3A, IFT88, and Ac-α-Tub protein expression and lung function indices of silicosis.

## Results

### SiO_2_ induces collagen deposition and myofibroblast differentiation *in vivo* and *in vitro*

**Figure 1:**
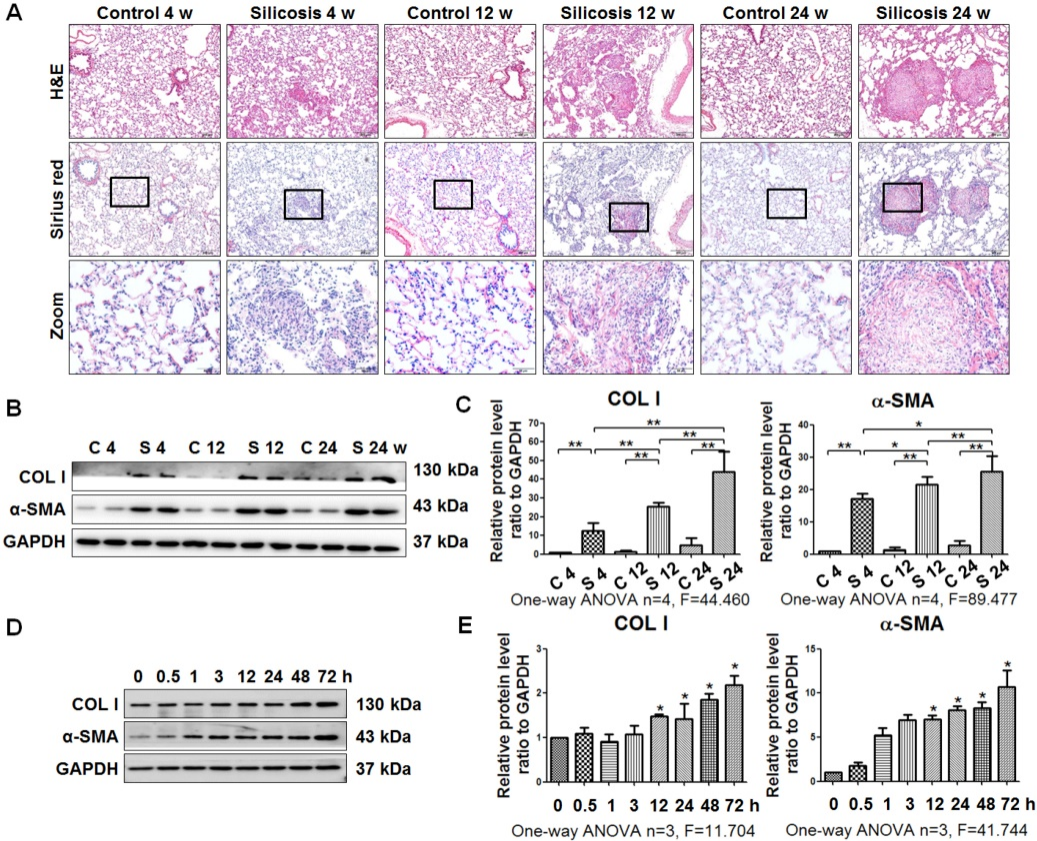
** Experimental silicotic rat and myofibroblast differentiation models established by SiO_2_ stimulation. (A)** H&E staining for pathological observation and Sirius Red staining of collagen in lung sections from rats at 4, 12, and 24 weeks after exposure to pure gas or a SiO_2_ mixture gas. Representative images are shown (n=10 per group, scale bars=200 and 50 μm). **(B)** Western blot indicating that SiO_2_ increased the expression of COL I and α-SMA in lung tissue of rats. GAPDH was used as a loading control (n=4). **(C)** Densitometric analyses suggesting that SiO_2_ induced COL I and α-SMA expression in rat lung tissue in a time-dependent manner. *P<0.05; **P<0.01. **(D)** Western blot showing the effects of SiO_2_ (50 μg/cm^2^) on expression of COL I and α-SMA in MRC-5 fibroblasts. GAPDH was used as a loading control (n=3). **(E)** Densitometric analyses suggesting that SiO_2_ induced COL I and α-SMA expression in MRC-5 fibroblasts in a time-dependent manner. *P<0.05 vs. SiO_2_ stimulation at 0 h. Data are the mean±SD. Statistical analysis was performed using one-way ANOVA and SPSS 20.0.

As described previously 20, 22, silicotic lesions, interstitial fibrosis, and collagen deposition depend on the time of exposure to silica in rats (Figure 1A). Accordingly, the levels of COL I and α-SMA were upregulated in silicotic rats in a time-dependent manner (Figure 1B, C). In addition, SiO_2_-stimulated MRC-5 fibroblasts *in vitro* exhibited increases in the levels of COL I and α-SMA in a time-dependent manner (Figure 1D, E).

### Primary cilia are lost during SiO_2_-induced myofibroblast differentiation

**Figure 2:**
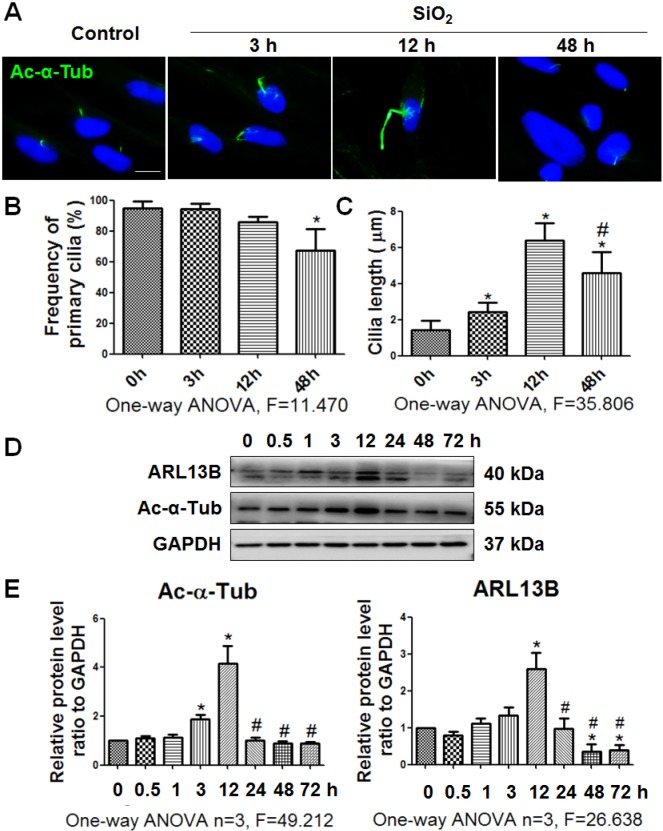
** Observation of primary cilia in MRC-5 fibroblasts treated with SiO_2_. (A)** IF showing changes in primary cilia of MRC-5 fibroblasts treated with SiO_2_. Primary cilia were labelled with an anti-Ac-α-Tub antibody. Scale bar=5 μm. Quantification of the frequency **(B)** and length **(C)** of primary cilia under the indicated conditions (mean±SD, n=3, >100 cells/experiment). **(D)** Western blot showing the effects of SiO_2_ (50 μg/cm^2^) on expression of ARL13B and Ac-α-Tub in MRC-5 fibroblasts. GAPDH was used as a loading control (n=3). **(E)** Densitometric analyses indicating that SiO_2_ induced ARL13B and Ac-α-Tub expression in MRC-5 fibroblasts. *P<0.05 vs. SiO_2_ stimulation at 0 h; #*P*<0.05 vs. SiO_2_ stimulation at 12 h. Data are the mean±SD. Statistical analysis was performed using one-way ANOVA and SPSS 20.0.

In the present study, we used Ac-α-Tub as a cilium marker 6 to reveal changes of primary cilia during myofibroblast differentiation induced by SiO_2_. The length of primary cilia was increased gradually until 12 h of SiO_2_ treatment compared with 0 h. However, after 48 h, most primary cilia were lost (Figure 2A-C). In addition, coexpression of Ac-α-Tub with other cilium markers (ARL13B, and γ-Tubulin 5) indicated a decrease of primary cilia in MRC-5 fibroblasts treated with silica for 48 h (Figure S2). The levels of Ac-α-Tub and ARL13B in MRC-5 fibroblasts also showed similar trends in western blots (Figure 2D, E).

### Primary cilia are lost in silicotic lesions of silica-exposed rats and silicotic patients

**Figure 3:**
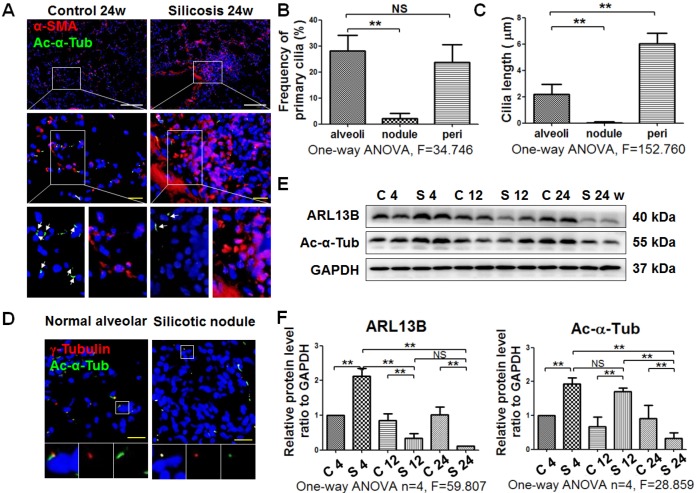
** Observation of primary cilia in silicotic lung tissue. (A)** IF staining of primary cilia (anti-Ac-α-Tub, arrow) in myofibroblasts (anti-α-SMA) in normal alveolar and silicotic nodules. Scale bars=200 and 30 μm. Quantification of the frequency **(B)** and length **(C)** of primary cilia under the indicated conditions (mean SD, n=3, >five views/experiment). **(D)** IF staining of primary cilia in normal and silicotic lungs using anti-Ac-α-Tub (green) and anti-γ-tubulin (red) antibodies. Scale bar=30 μm. **(E)** Western blot showing the effects of SiO_2_ inhalation on expression of ARL13B and Ac-α-Tub in rat lung tissue. GAPDH was used as a loading control (n=4). **(F)** Densitometric analyses of ARL13B and Ac-α-Tub expression in rat lung tissue. **P<0.01. Data are the mean±SD. Statistical analysis was performed using one-way ANOVA and SPSS 20.0.

IF staining showed a decrease of primary cilia (marked by anti-Ac-α-Tub green fluorescence) in silicotic lesions accompanied by an increase of α-SMA-positive cells (Figure 3A). The frequency and length of primary cilia were decreased in silicotic nodules compared with alveoli walls (Figure 3B, C). This result was confirmed by Ac-α-Tub IHC staining (Figure S3), costaining of γ-Tubulin and Ac-α-Tub (Figure 3D), and western blot analysis of Ac-α-Tub and ARL13B in rats (Figure 3E, F). We also found that primary cilia were consistently long in pericarcinomatous and perinodule lung tissues, and lost in cellular and fibrous silicotic nodules with rich collagen deposition and α-SMA IHC staining (Figure 3G-J).

**Figure 4:**
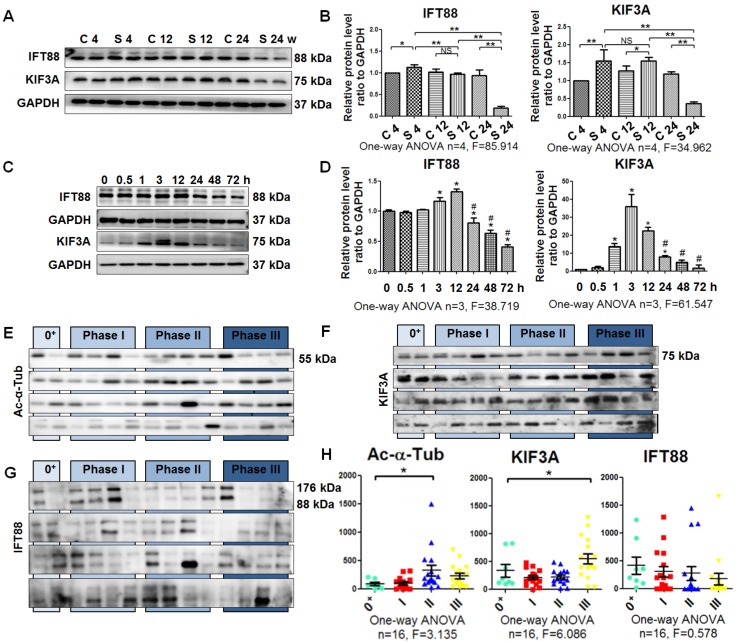
** Detection of ciliary proteins. (A, B)** Western blot showing the effects of SiO_2_ stimulation on expression of IFT88 and KIF3A proteins in rat lung tissue. GAPDH was used as a loading control. **(C, D)** Western blot showing the IFT88 and KIF3A expression in MRC-5 fibroblasts induced by SiO_2_. *P<0.05 vs. SiO_2_ stimulation at 0 h. ^#^*P*<0.05 vs. SiO_2_ stimulation at 12 h. Data are the mean±SD. Statistical analysis was performed using one-way ANOVA and SPSS 20.0. **(E-G)** Western blot showing the expression of Ac-α-Tub, IFT88, and KIF3A proteins in BALF of silicotic patients. The relative density was normalised to the protein concentration. **(H)** Densitometric analyses of Ac-α-Tub, IFT88, and KIF3A protein expression in BALF. *P<0.05; **P<0.01. Data are the mean±SD. Statistical analysis was performed using one-way ANOVA and SPSS 20.0.

Next, we quantified cilium-related proteins including kinesin-2 motor KIF3A and anterograde axoplasmic cargo transporter IFT88 23. The levels of KIF3A and IFT88 were increased in rats exposed to silica for 4 weeks and decreased in the 24-week silicosis group compared with their control groups. Accordingly, expression of KIF3A and IFT88 in SiO_2_-induced myofibroblasts was significantly upregulated at 12 h and then decreased until 72 h (Figure 4A-D). Taken together, these results showed that the emergence of a myofibroblast phenotype during silicosis was accompanied by a loss of primary cilia.

### Shedding of ciliary proteins KIF3A and Ac-α-Tub induced by SiO_2_

It has been reported that primary cilia shed into urine during AKI, and detection of primary cilia fragments or ciliary proteins in urine can be useful to evaluate the severity of kidney injury 8. Therefore, we hypothesised that primary cilia may be shed into the alveolar cavity during silicosis. The levels of IFT88, KIF3A, and Ac-α-Tub were measured in BALF of silicotic patients. We found that Ac-α-Tub and KIF3A in BALF were significantly increased in silicotic patients in phase II and stage III, respectively. However, the level of IFT88 was not changed significantly (Figure 4E-H). Furthermore, we found that the levels of Ac-α-Tub and KIF3A were increased in BALF of silicotic rats and culture supernatants of SiO_2_-stimulated MRC-5 fibroblasts (Figure S4). The relationships between primary ciliary markers and lung function indices are listed in Table 1. The level of KIF3A was significantly negatively correlated with VC, FVC, and DLco (*r*=-0.302, *P*=0.031; *r*=-0.300, *P*=0.033; *r*=-0.311, *P*=0.026, respectively). These results indicated that the shedding of primary cilia may be induced by silica.

### *KIF3A* knockdown accelerates myofibroblast differentiation induced by silica

Some recent studies have demonstrated that the role of primary cilia may be associated with a biphasic mechanism where the first phase of myofibroblast differentiation requires a fibrotic signalling pathway regulated by primary cilia and the second phase appears to be specific to cell types, which may involve loss or reduction in the length of primary cilia. These mechanisms result in sustained myofibroblast activation and subsequent fibrosis 5, 10. In line with a previous report 6, silencing of *KIF3A* before silica treatment inhibited myofibroblast differentiation (Figure S5).

**Figure 5:**
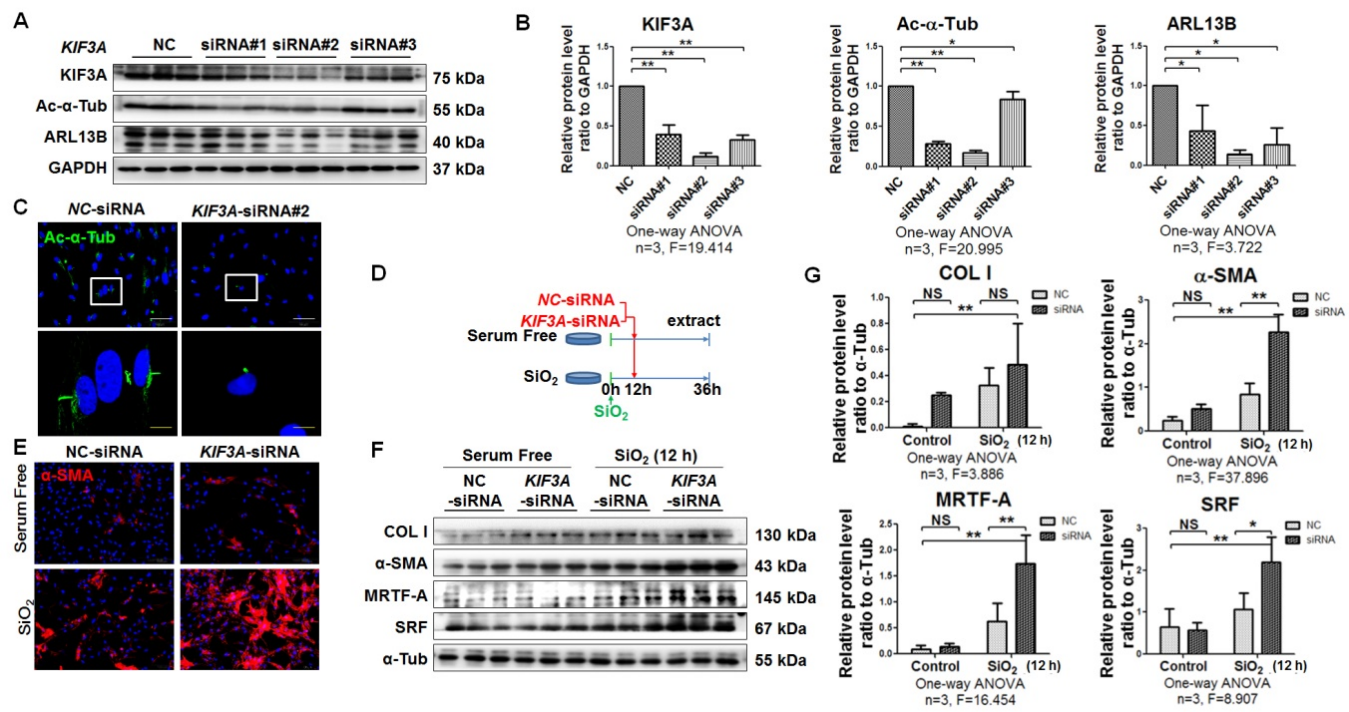
***KIF3A* knockdown increases α-SMA-positive myofibroblasts among SiO_2_-activated MRC-5 fibroblasts. (A)** Western blot showing the effects of *NC*-siRNA and *KIF3A*-siRNA on expression of KIF3A, Ac-α-Tub, and ARL13B proteins in MRC-5 fibroblasts. GAPDH was used as a loading control (n=3). **(B)** Densitometric analyses of KIF3A, Ac-α-Tub, and ARL13B protein expression in MRC-5 fibroblasts. *P<0.05; **P<0.01. Data are the mean±SD. Statistical analysis was performed using one-way ANOVA and SPSS 20.0. **(C)** IF assay showing primary cilia in MRC-5 fibroblasts treated with *NC*-siRNA or *KIF3A*-siRNA. Primary cilia were labelled with an anti-Ac-α-Tub antibody. Scale bar=25 and 5 μm. **(D)** Treatment regimen of *KIF3A* knockdown in MRC-5 fibroblasts. MRC-5 fibroblasts were stimulated with SiO_2_ or serum-free medium (n=3 per group) for 12 h, and then transfected with *NC*-siRNA or *KIF3A*-siRNA until 36 h. **(E)** Expression of α-SMA in MRC-5 fibroblasts measured by IF. Scale bar=100 μm. **(F, G)** Western blot and densitometric analyses of the effects of *NC*-siRNA and *KIF3A*-siRNA on expression of COL I, α-SMA, MRTF-A and SRF proteins in MRC-5 fibroblasts with or without SiO_2_ stimulation. α-Tub was used as a loading control (n=3). *P<0.05; **P<0.01. Data are the mean±SD. Statistical analysis was performed using one-way ANOVA and SPSS 20.0.

Therefore, we first treated MRC-5 fibroblasts with SiO_2_ for 12 h and then silenced *KIF3A* for another 24 h. *KIF3A* silenced by specific siRNA resulted in loss of cilia and reduced ciliary proteins (Figure 5A-D). Compared with NC-siRNA transfection,* KIF3A*-siRNA transfection did not change the levels of α-SMA or COL I expression in unstimulated MRC-5 fibroblasts. However, *KIF3A*-siRNA transfection after SiO_2_ stimulation significantly increased the expression of α-SMA, SRF, and MRTF-A (Figure 5E-G). Similarly, knockdown of *IFT88* in SiO_2_-stimulated MRC-5 fibroblasts significantly exacerbated myofibroblast differentiation induced by SiO_2_ (Figure S6). These data suggested that cilium loss promotes or facilitates myofibroblast differentiation.

### *KIF3A* silencing disrupts SHH signalling

**Figure 6:**
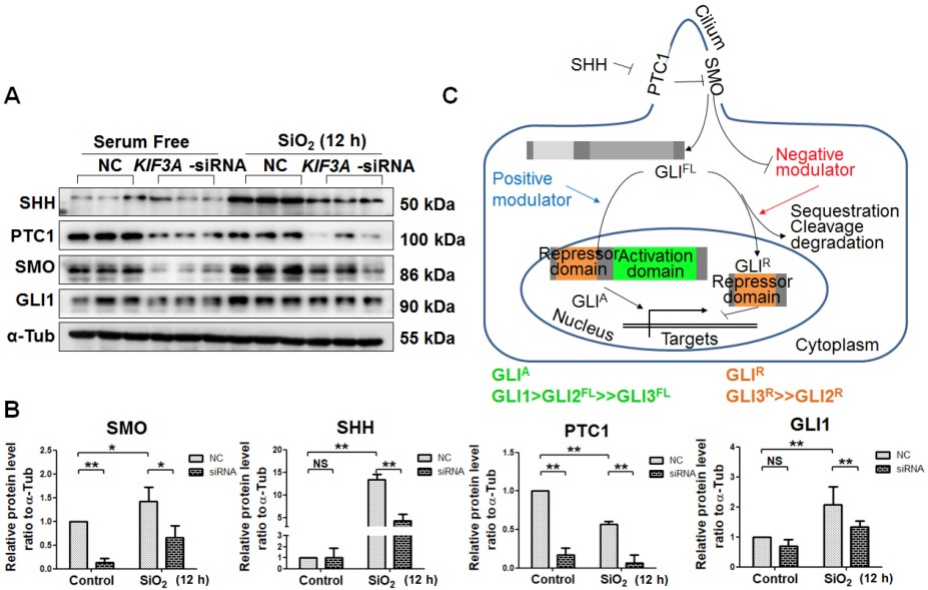
** Detection of SHH signalling proteins in MRC-5 fibroblasts in response to SiO_2_ stimulation and* KIF3A* knockdown. (A, B)** Western blot and densitometric analyses of SHH, PTC1, SMO, and GLI1 proteins in MRC-5 fibroblasts treated with SiO_2_ for 12 h together with* KIF3A* knockdown until 36 h. The relative density was normalised to α-Tub. Bar graphs are the means±SD of three separate experiments. *P<0.05; **P<0.01. Data are the mean±SD. Statistical analysis was performed using one-way ANOVA and SPSS 20.0. **(C)** Schematic of the primary cilia influence on SHH signalling. Bidirectional transcriptional factors regulate the transcription of target genes.

Activation of SHH signalling, which is necessary for myofibroblast differentiation and proliferation, is influenced by primary cilia 24, 25. In the present study, SiO_2_ treatment increased the levels of SHH, SMO, and GLI1 accompanied by a reduced level of PTC1. *KIF3*A silencing decreased the levels of SHH, PTC1, SMO, and GLI1 after SiO_2_ treatment for 24 h (Figure 6A-C). These results indicated that the ligands and receptors of SHH signalling, including SHH, PTC1, and SMO, were disrupted because of the loss of primary cilia induced by *KIF3A* silencing. In addition, the level of GLI1, the major transcription factor of SHH signalling, was decreased by silencing *KIF3A*.

### *KIF3A* silencing promotes GLI2^FL^ to bind and activate *SRF* transcription

**Figure 7:**
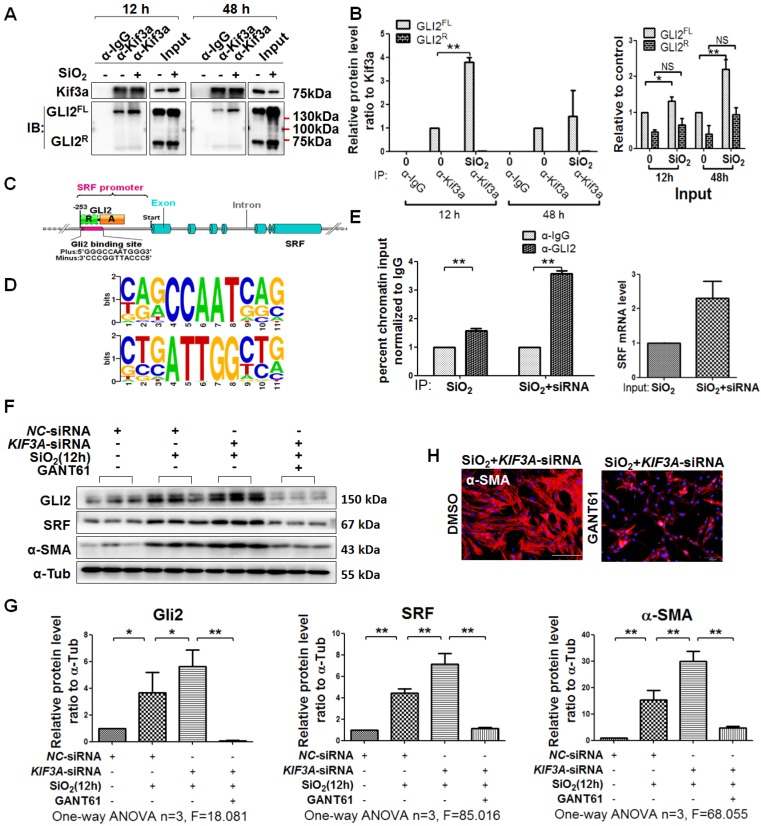
***KIF3A*-siRNA promotes Gli2 enrichment of the *SRF* promoter in MRC-5 cells. (A, B)** Anti-KIF3A immunoprecipitates (IPs) from whole cell lysates of MRC-5 cells treated with SiO_2_ for 12 and 48 h. Pulled-down and input lysates were examined for expression of KIF3A and GLI2 by western blot and densitometric analyses. The relative densities of pulled-down and input lysates were normalised to KIF3A and the control, respectively. Data are presented as means±SD. **(C)** Schematic of the distribution of GLI2-binding sites (GBSs) within the *SRF* promoter. **(D)** Consensus sequence of GBS GLI2-binding sites. **(E)** ChIP-qPCR with α-GLI2 and α-IgG pull-down and qPCR amplification against *SRF*. Genomic DNA from MRC-5 cells treated with SiO_2_ (left panel) and SiO_2_+*KIF3A* siRNA (right panel) immunoprecipitated with α-GLI2 and non-specific α-IgG antibodies was used for qPCR to assess the fold enrichment of the respective gene promoters in GLI2-IP DNA over IgG-IP for each gene. Fold enrichments are the averages of three independent experiments. Data are presented as means±SD. **(F, G)** Western blot analysis and quantification of Gli2, SRF, and α-SMA in MRC-5 cells treated with SiO_2_ for 12 h together with* KIF3A* knockdown until 36 h of cotreatment with GANT61. The relative density was normalised to α-Tub. Bar graphs are the means±SD of three separate experiments, *P<0.05, **P<0.01. Data are the mean±SD. Statistical analysis was performed using one-way ANOVA and SPSS 20.0. (H) IF assay showing expression of α-SMA in MRC-5 cells treated with SiO_2_ for 12 h together with* KIF3A* knockdown until 36 h of cotreatment with GANT61. Scale bar=100 μm.

It has been reported that KIF3A interacts with GLI proteins 18. We also found that GLI2^FL^ protein formed a complex with KIF3A, and the binding between GLI2^FL^ and KIF3A was increased by SiO_2_ stimulation (Figure 7A, B). GLI2^FL^ was increased with silicosis progression *in vivo* and *in vitro* (Figure S7). Next, we used an online tool (http://rna.sysu.edu.cn) to identify putative GLI-binding sites (GBSs) in the genomic sequence adjacent to the transcription start site of the *SRF* gene to determine whether GLI2 binds to the *SRF* promoter and directly activates its transcription. We found a putative GBS (Figures 7C, D and S8) within the -253 genomic regions relative to the 5′ initiation site of *SRF*. A ChIP assay showed that GLI2 bound to *SRF* (Figure 7E). Taken together, these results identified *SRF*, a transcription factor of α-SMA 26, as a novel direct target gene of GLI2^FL^. Furthermore, treatment with GANT61, an inhibitor of GLI2, markedly blocked the SiO_2_+*KIF3A*-siRNA-induced upregulation of GLI2^FL^ along with downregulation of SRF and α-SMA (Figure 7F-H).

### *KIF3A* knockdown reduces GLI3^R^ to decrease the inhibitive effect of GLI3^R^ on *ACTA2*

**Figure 8:**
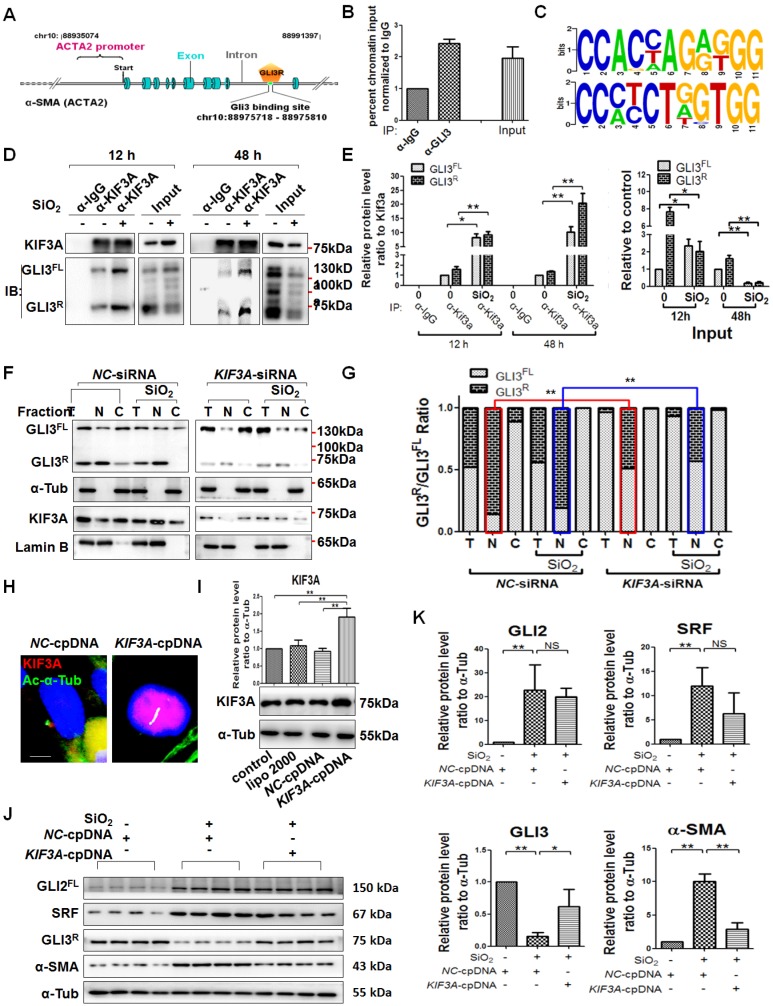
***KIF3A* knockdown reduces GLI3^R^ to decrease the inhibitive effect of GLI3^R^ on *ACTA2*. (A)** Schematic of the distribution of GLI3-binding sites within the *ACTA2* promoter. **(B)** ChIP-qPCR with α-GLI3 and α-IgG pull-down and qPCR amplification against *ACTA2*. Genomic DNA from untreated MRC-5 cells immunoprecipitated with α-GLI3 and non-specific α-IgG antibodies was used for qPCR to assess the fold enrichment of the respective gene promoters in GLI3-IP DNA over IgG-IP for each gene. **(C)** Consensus sequence of GBS GLI2-binding sites. **(D, E)** Anti-KIF3A immunoprecipitates (IPs) from whole cell lysates of MRC-5 cells treated with SiO_2_ for 12 and 48 h. Pulled-down and input lysates were examined for expression of KIF3A and GLI3 by western blotting and densitometric analyses. The relative densities of pulled-down and input lysates were normalised to KIF3A and the control, respectively. Data are presented as means±SD. (F) MRC-5 cells were treated with *NC*-siRNA, SiO_2_+*NC*-siRNA,* KIF3A*-siRNA, or SiO_2_+*KIF3A*-siRNA. Kif3a, GLI3^FL^, and GLI3^R^ proteins were detected in total (T), nuclear (N), and cytoplasmic (C) fractions. Lamin B and α-Tub were used as loading controls of nuclear and cytoplasmic proteins, respectively. The ratio of GLI3^FL^ and GLI3^R^ under each condition in **(F)** is plotted in **(G)**, **P<0.01. **(H, I)** IF assay and western blotting verified the effect of the *KIF3A*-cpDNA vector. Scale bar=3 μm. Bar graphs are the means±SD of three separate experiments. *P<0.05; **P<0.01. Data are the mean±SD. Statistical analysis was performed using one-way ANOVA and SPSS 20.0. **(J, K)** MRC-5 cells were treated with *NC*-cpDNA, SiO_2_+*NC*-cpDNA, or SiO_2_+*KIF3A*-cpDNA. The levels of GLI2^FL^, SRF, GLI3^R^, and α-SMA proteins were determined by western blotting and densitometric analyses. A-Tub was used as a loading control (n=3). *P<0.05; **P<0.01. Data are the mean±SD. Statistical analysis was performed using one-way ANOVA and SPSS 20.0.

As described above, GLI3 exists as both a full-length form (GLI^FL^) as a transcriptional activator and a proteolytically processed form as a transcriptional repressor (GLI^R^) that serves exclusively as a strong repressor of target genes 16, 17. First, we matched the transcriptional repressor GLI3^R^ to *ACTA2* (α-SMA-encoding gene), and ChIP results showed that GLI3 bound to ACTA2, suggesting that GLI3^R^ directly inhibited α-SMA transcription (Figures 8A-C and S8). KIF3A motor protein is required for GLI3^FL^ proteolysis to GLI3^R^ 18. The results of CoIP demonstrated that GLI3^FL^ and GLI3^R^ proteins formed a complex with KIF3A, and the binding was even high at 48 h of SiO_2_ stimulation when the levels of GLI3^FL^ and GLI3^R^ were decreased significantly (Figure 8D, E). *KIF3A* silencing decreased the levels of GLI3^R^ (Figure 8J, K), especially in the nuclear fraction (Figure 8F, G), indicating that the inhibitive effect of GLI3^R^ on *ACTA2* could be reversed by reducing KIF3A. Furthermore, we found that *KIF3A* overexpression significantly inhibited the upregulation of α-SMA induced by SiO_2_, and the level of GLI3^R^ protein expression was upregulated correspondingly (Figure 8 J, K). However, the levels of GLI2^FL^ and SRF were not changed significantly in SiO_2_-stimulated *KIF3A-cpDNA* cells compared the SiO_2_-stimulated *NC-cpDNA* cells.

### KIF3A stabilizes GLI3^R^

**Figure 9:**
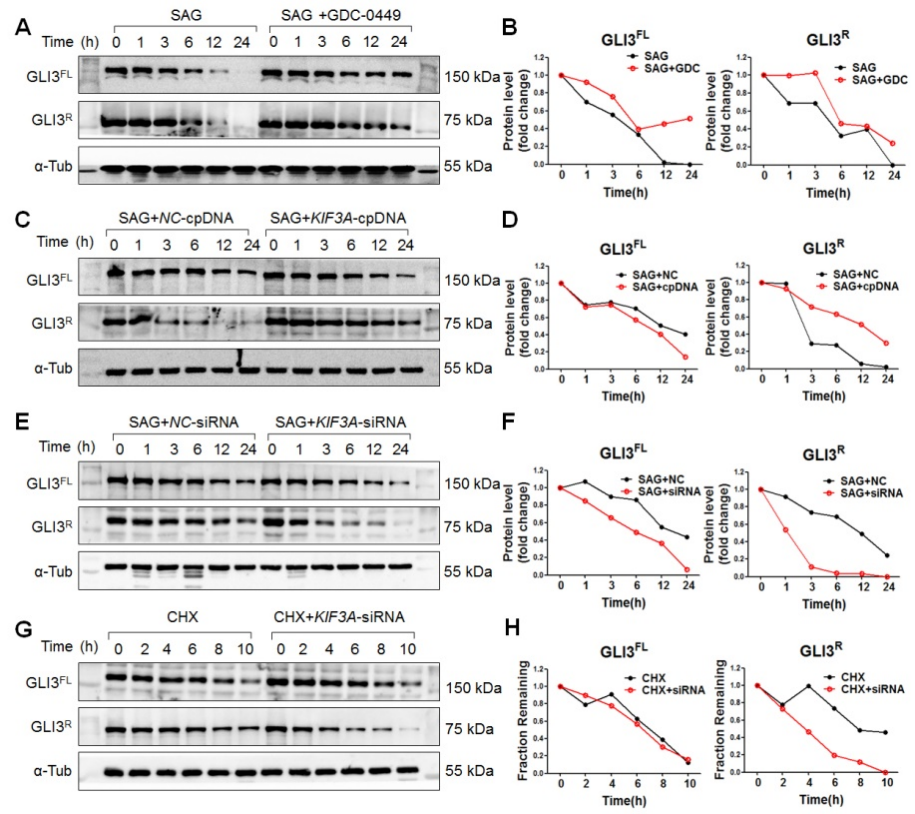
** KIF3a increases the stability of transcriptional repressor Gli3^R^ but not Gli3^FL^ in response to SMO activation. (A, B)** Gli3^FL^ and Gli3^R^ protein levels in MRC-5 cells treated with SAG alone or SAG+GDC-0449 for the indicated periods of time. **(C, D)** Gli3^FL^ and Gli3^R^ protein levels in MRC-5 cells treated with SAG+*NC*-cpDNA or SAG+*KIF3A*-cpDNA for the indicated periods of time. **(E, F)** Gli3^FL^ and Gli3^R^ protein levels in MRC-5 cells treated with SAG+*NC*-siRNA or SAG+*KIF3A*-siRNA for the indicated periods of time. **(G, H)** Gli3^FL^ and Gli3^R^ protein levels in MRC-5 cells treated with CHX alone or CHX+*KIF3A*-siRNA for the indicated periods of time. Levels of Gli3^FL^ and Gli3^R^ protein were measured by western blotting. α-Tub was used as a loading control. Scatter diagrams are the means±SD of three separate experiments.

Because SHH signalling was blocked by *KIF3A* silencing under SiO_2_ treatment, we used SAG (a SMO agonist) to explore the role of KIF3A in GLI3^R^ under activated SHH signalling. As shown in Figure 9, *KIF3A* cpDNA increased the level of GLI3^R^, whereas *KIF3A*-siRNA decreased the level of GLI3^R^. CHX chase experiments showed that *KIF3A-*siRNA accelerated the degradation of GLI3^R^ induced by CHX (Figure 9G, H). We also found that destabilisation of GLI3^R^ was not influenced by *IFT88* silencing (Figure S5E, F).

## Discussion

In the present study, we found a decrease of primary cilia during silicosis, which is different to previous studies 5, 6. In human IPF tissue samples, the distribution and increase of primary cilia are found in alveolar epithelial cells, fibroblasts, and endothelial cells compared with normal human lung tissue 5. Ciliated fibroblasts are enriched in areas of myocardial injury 6. In addition, we found that primary cilia and ciliary proteins increased at first and then decreased in rats and MRC-5 fibroblasts exposed to silica. However, the elongation of primary cilia is almost located in the margin of fibrotic lesions in IPF 5 or in ischemia/reperfusion models 6. These morphological observations support our results in silicotic rats. Recently, a review of primary cilia in fibrosis may explain the difference, which indicated that the role of the primary cilium may be associated with a biphasic mechanism and has cell-, tissue-, and disease-specific mechanisms 10.

Most studies have recognised changes in the primary length, including elongation and shortening caused by the reabsorption or disruption of cilia, as adaptive responses to environmental stimuli 8. In IPF 5, injured myocardium 6, kidneys 27, 28, and pancreatic ducts 13, 29, the cilium length increases significantly in response to cellular injury. However, primary cilium loss contributes to a switch from reversible to irreversible kidney injury in the unilateral ureteral occlusion (UUO) model 7. Additionally, detection of the primary cilium length and fragments in urine can be useful to evaluate the severity of kidney diseases 8. We also found that the levels of Ac-α-Tub and KIF3A were increased in BAFL and culture supernatants of SiO_2_-stimulated MRC-5 fibroblasts, indicating that organ fibrosis may be accompanied by primary cilium shedding.

Primary cilia are required for myofibroblast differentiation regulated by coordination and transduction of fibrotic morphogen signalling pathways 10. Many fibrotic signalling pathways, including TGF-β1 30, Wnt 7, and SHH 31 localize and signal in the ciliary membrane. Cilia are required for TGF-β1-triggered myofibroblast activation and contractile functions, which are blocked by siRNA-mediated downregulation of *PC1* or *KIF3A* during cardiac fibrosis 6. The disruption of primary cilia inhibits myofibroblast differentiation from adipose progenitors induced by TGF-β1 32. In the present study, we also found that transfection of *KIF3A*-siRNA into MRC-5 fibroblasts inhibited myofibroblast differentiation induced by silica. However, shortening or loss of primary cilia can promote and accelerate myofibroblast differentiation. During epithelial-myofibroblast transition (EMyT), initial cilium growth is followed by complete deciliation 9. In addition, non-ciliated endothelial cells undergo endothelial-mesenchymal transition induced by fluid shear stress, which is prevented by blocking TGF-β1 signalling, overexpression of *Klf4*, or rescue of the primary cilium 33. In the present study, we treated MRC-5 fibroblasts with silica for 12 h and then knocked down *KIF3A* or *IFT88*, which enhanced myofibroblast differentiation characterized by increased levels of α-SMA, SRF, and MRTF-A. However, silencing *KIF3A* inhibited myofibroblast differentiation. These results indicate that primary cilia have a biphasic mechanism for myofibroblast differentiation. During the myofibroblast transition, key morphogen signalling pathways, including TGF-β1 30, Wnt 7, and SHH 31, have been implicated in driving or sustaining pathological myofibroblast transition by the primary cilium. In addition, shortening or losses of primary cilia are necessary to maintain the myofibroblast phenotype 10.

Furthermore, we focused on the SHH signalling pathway regulated by primary cilia. Our previous study showed that activation of SHH signalling has a major role in silicosis 14. In the present study, silica activated SHH, SMO, GLI1, and GLI2^FL^ in MRC-5 fibroblasts and caused decreases of PTC1 and GLI3^R^. It has been reported that KIF3A, the anterograde IFT motor, is required for both formation of the GLI activator and proteolytic processing of GLI3 34. Moreover, we found that reducing KIF3A promoted myofibroblast differentiation by increasing CLI2^FL^-*SRF* and decreasing the inhibitory effect of CLI3^R^ on *ACTA2*. During activation of SHH signalling induced by SAG, *KIF3A*-cpDNA increased the level of GLI3^R^ and *KIF3A*-siRNA decreased the level of GLI3^R^. These results indicate that KIF3A is required for the formation of GLI3^R^, which provide a possible explanation for why deciliation of the cilium-associated pathway represents dysregulation rather than overall inhibition of fibrogenic signalling. Loss of KIF3A destabilised GLI3^R^, which was unable to counteract activator-formed GLIs, resulting in the transcriptional activation of target genes in MRC-5 fibroblasts after SiO_2_ stimulation.

**Figure 10:**
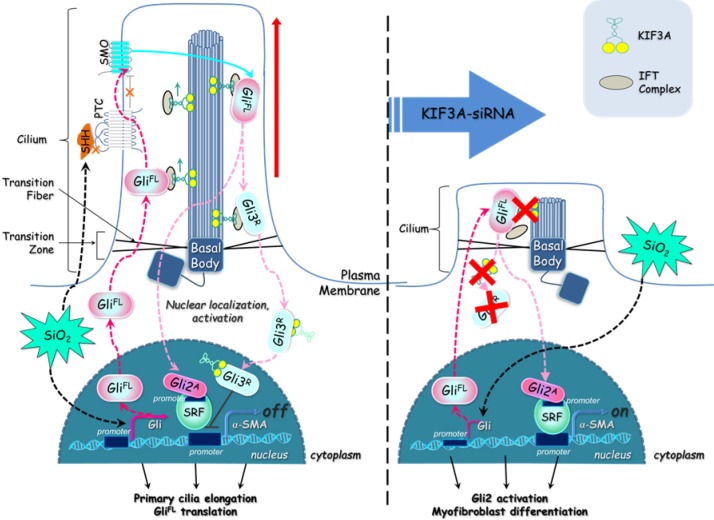
** Defective *KIF3A* perturbs ciliogenesis and promotes myofibroblast differentiation.** The major processes underlying KIF3A regulation of primary cilia and GLI3 that influences α-SMA expression. For further explanation, see the Discussion section.

Taken together, these results demonstrate that silica activates SHH signalling via enlargement of primary cilia and decreases KIF3A, resulting in the loss of primary cilia and accelerating myofibroblast differentiation by inhibition of GLI3^R^ (Figure 10). This study provides a unique insight into the loss of primary cilia and the formation of myofibroblasts during silicosis.

## Supplementary Material

Supplementary figures and table.

## Figures and Tables

**Table 1:** Correlations between primary ciliary markers and lung function indices in silicosis patients.

	VC		FVC		FEV_1_		FEV_1_/FVC		DL_CO_		RV		RV/TLC	
	*r*	*P*	*r*	*P*	*r*	*P*	*r*	*P*	*r*	*P*	*r*	*P*	*r*	*P*
KIF3A	-0.302*	0.031	-0.300*	0.033	-0.251	NS	-0.027	NS	-0.311*	0.026	-0.212	NS	0.039	NS
IFT88	0.050	NS	0.055	NS	0.062	NS	0.034	NS	-0.027	NS	0.209	NS	0.070	NS
Ac-α-Tub	-0.260	NS	-0.219	NS	-0.067	NS	0.252	NS	-0.221	NS	0.211	NS	0.034	NS

## Abbreviations

Ac-α-Tub: acetylated tubulin α
KIF3A: kinesin family member 3A
GLIs: Glioma-associated oncogene homologs
ITF88: intraflagellar transport 88
SHH: sonic hedgehog
PTC1: patched
SMO: smoothened
ECM: extracellular matrix
CWP: coal worker's pneumoconiosis
BALF: bronchoalveolar lavage fluid
IF: immunofluorescence
IHC: immunohistochemistry
CoIP: co-immunoprecipitation
ChIP: chromatin immunoprecipitation
GBS: GLI-binding sites
TSS: transcription start site
