# Supplementary material for: Silica Perturbs Primary Cilia and Causes Myofibroblast Differentiation during Silicosis by Reduction of the KIF3A-Repressor GLI3 Complex
Source: Theranostics. 2020 Jan 1;10(4):1719–32. doi: 10.7150/thno.37049 (PMC6993221; doi:10.7150/thno.37049)
Supplement: Supplementary file 1 — Supplementary figures and table. [file thnov10p1719s1.pdf]

Silica Perturbs Primary Cilia and Causes Myofibroblast Differentiation during Silicosis by Reduction of the KIF3A-Repressor GLI3 Complex

Shifeng Li[1], Zhongqiu Wei[2], Gengxu Li [2], Qiaodan Zhang[1], Siyu Niu[1], Dingjie Xu[3], Na Mao[4], Si Chen[5], Xuemin Gao[1], Wenchen Cai[4], Ying Zhu[1], Guizhen Zhang[1], Dan Li[1], Xue Yi[6], Fang Yang[1] and Hong Xu[1]*

1. Medical Research Center, Hebei Key Laboratory for Organ Fibrosis Research, North China University of Science and Technology, Tangshan, China

2. Basic Medicine College, North China University of Science and Technology, Tangshan, China

3. College of Traditional Chinese Medicine, North China University of Science and Technology, Tangshan, China

4. School of Public Health, North China University of Science and Technology, Tangshan, China

5. Department of Neurosurgery, Tangshan People's Hospital, Tangshan, China

6. Basic Medical College, Xiamen Medical Collage, Xiamen, China

*Corresponding author:

Hong Xu, Ph.D., M.D.

Medical Research Center

North China University of Science and Technology

No.21 Bohai Road, Tangshan city, Hebei 063000, China

Email: xuhong@ncst.edu.cn

Tel: +86-315-8816236

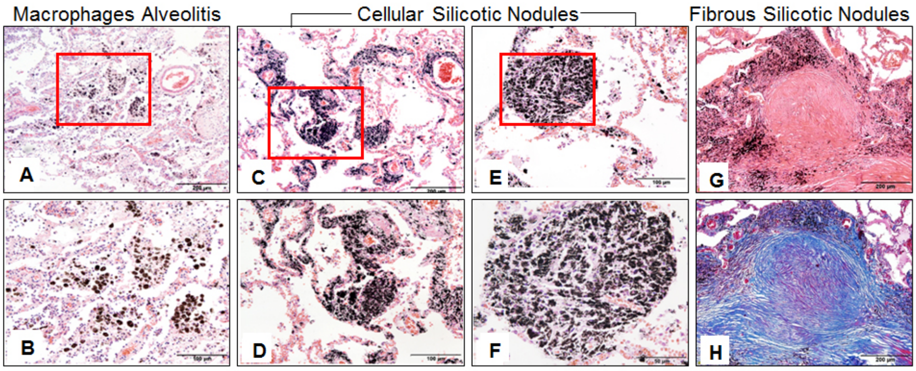

Figure S1 Morphology alterations in lungs of silicotic patients. (A, B) Macrophage alveolitis. There were many macrophages, neutrophils, and other inflammatory cells in the alveolar cavity, and black dust was seen in the lung tissue. (C–F) Cellular silicotic nodules indicating that cystic nodules formed in alveolar cavities of silicotic lungs. The nodular lesions were formed by aggregation of macrophages around vessels with a small amount of long fusiform cells and hyperplasia of collagen fibres. (G, H) Fibrous silicotic nodules, including glass-like nodules, and macrophages surrounding the silicotic nodules that contained dust particles. Abnormal hyperplasia of collagen fibres was arranged in concentric circles in the nodules, and the collagen in the centre of the nodules was often hyaline, showing glassy degeneration. Tissues in A–G were stained by H&E, and those in H were subjected to Masson's staining. Scale bar=200 μm; inset=100 μm.

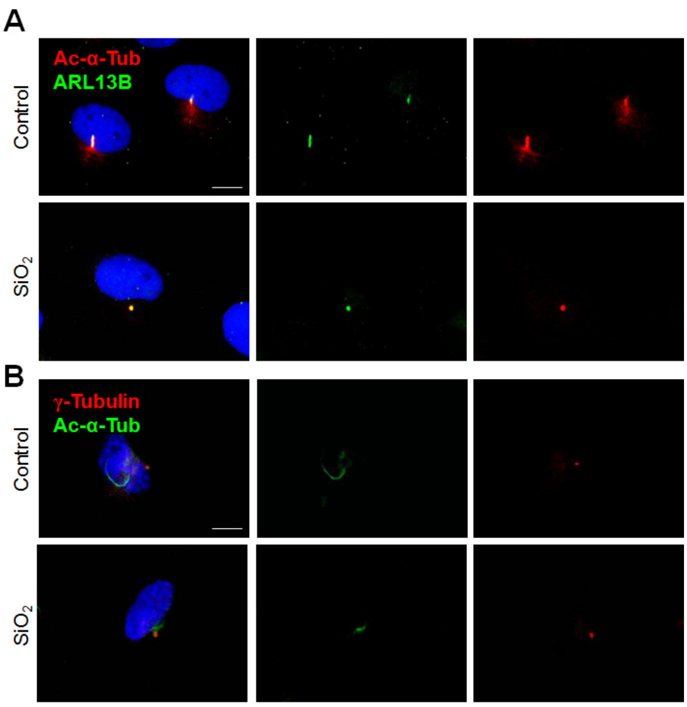

Figure S2 Primary cilia detected in MRC-5 cells treated with SiO_2. MRC-5 human embryonic lung fibroblasts were treated with serum-free medium or SiO_2 for 48 h. (A) Primary cilia were double labelled with Ac-α-Tub + ARL13B, markers of primary cilia. Coexpression of Ac-α-Tub + ARL13B was observed in primary cilia. (B) Primary cilia were double labelled with Ac-α-Tub + γ-Tubulin. Primary cilia were long in MRC-5 cells treated with serum-free medium and short in SiO_2-stimulated cells. Scale bar=10 μm.

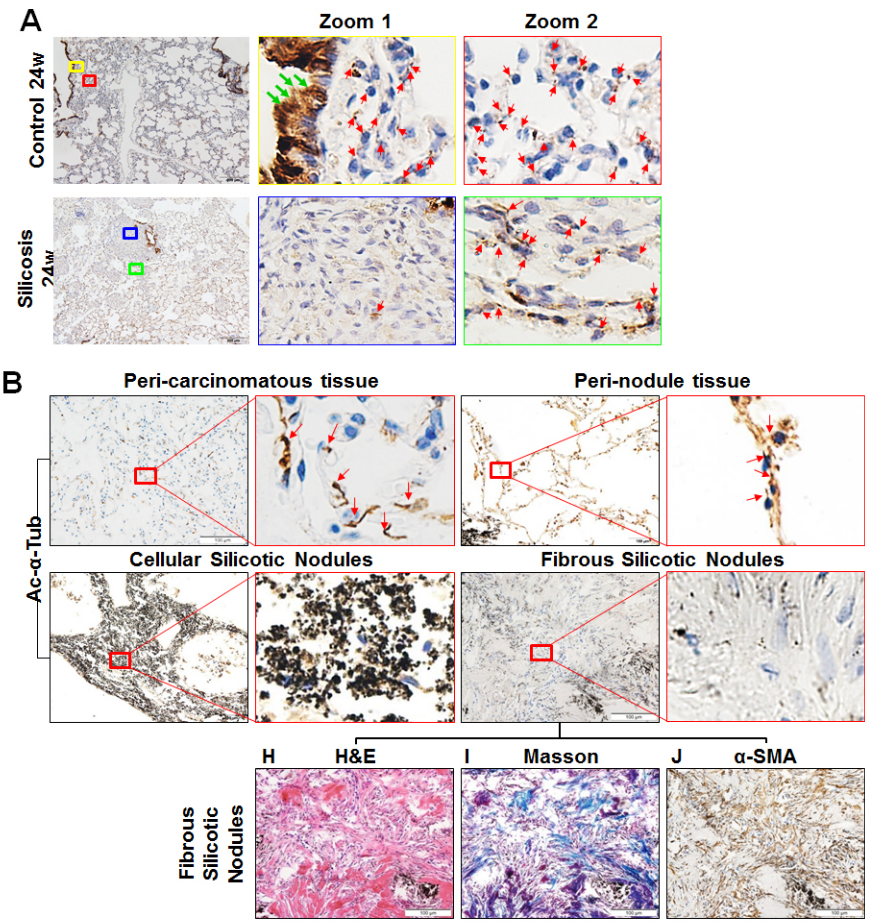

Figure S3 Primary cilia detected in lung tissue of silicotic rats. (A) Primary cilia in silicotic rat lung tissue were detected by Ac-α-Tub immunohistochemistry. Scale bar=100 μm. In 24-week control rats, primary cilia were observed in bronchial ciliary epithelial cells of the bronchia (yellow square, green arrow) and various cell types (alveolar epithelial cells and fibroblasts) in the alveolar wall (red square, red arrow). In 24-week silicotic rats, primary cilia were undetectable in silicotic nodules (blue square, red arrow), but were observed in alveolar wall cells around silicotic nodules (green square, red arrow). (B) Observation of primary cilia in lung tissue of silicotic patients. Primary cilia (red arrow), marked by Ac-α-Tub, in lung tissue of silicotic patients were measured by IHC. Scale bar=100 μm. Fibrous silicotic nodules were confirmed by H&E staining, Masson staining, and IHC of α-SMA.

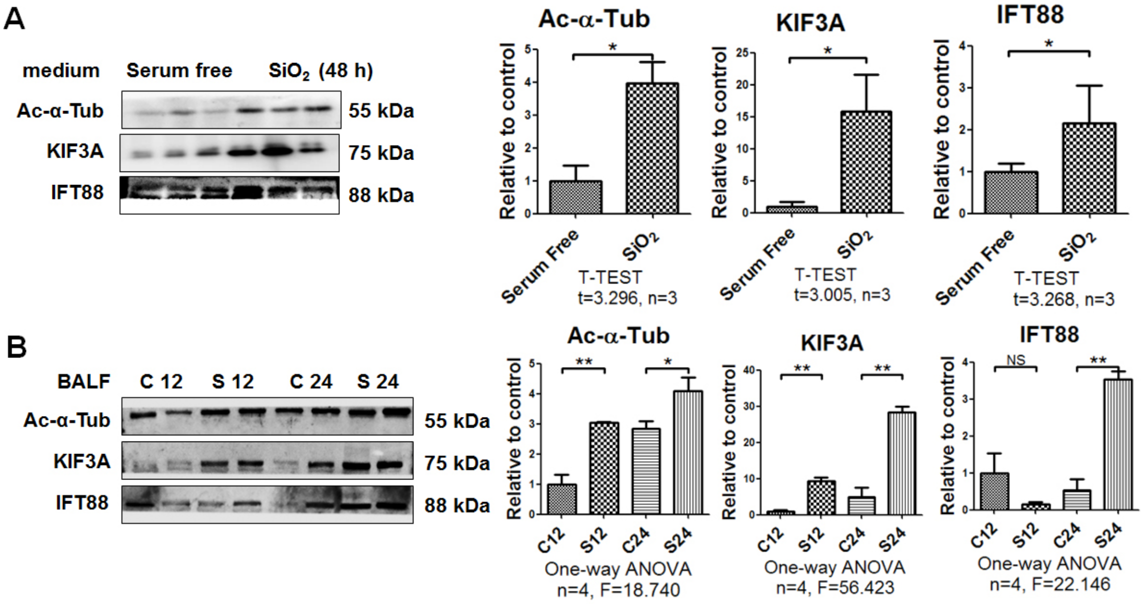

Figure S4 Primary cilia shed into BALF of rats and the culture supernatant of MRC-5 cells treated with SiO_2. (A) Levels of Ac-α-Tub, KIFA3, and IFT88 proteins in culture supernatants of MRC-5 cells treated with SiO_2 were measured by western blotting (n=3). Bar graphs are means±SD. Statistical analysis was performed using the t-test and SPSS 20.0. (B) Levels of Ac-α-Tub, KIF3A, and IFT88 proteins in BALF of silicotic rats were measured by western blotting (n=4). Bar graphs are the means±SD. Statistical analysis was performed using one-way ANOVA and SPSS 20.0.

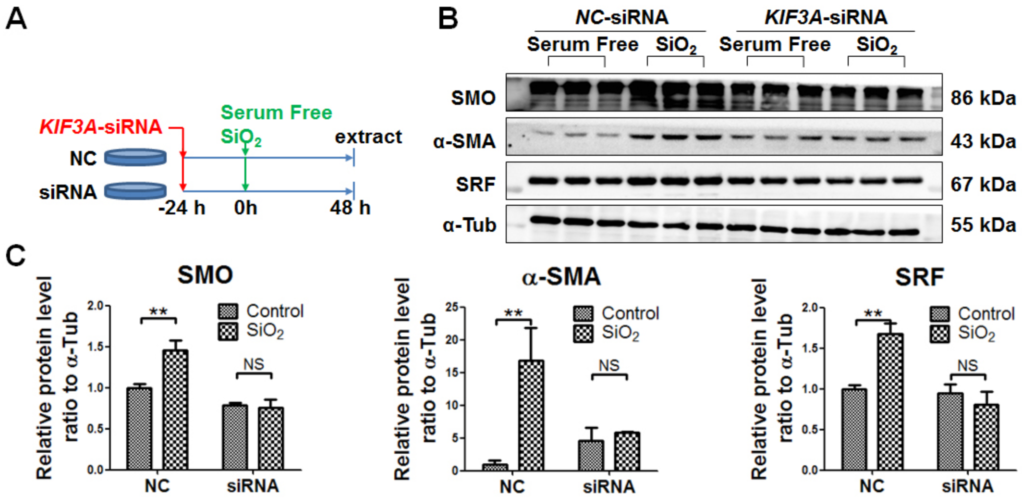

Figure S5 Primary cilia are required for myofibroblast activation. (A) Treatment regimen for *KIF3A* knockdown in MRC-5 cells. MRC-5 cells were transfected with siRNA for 24 h before stimulation with SiO_2 or serum-free medium for another 48 h (n=3). (B, C) Western blotting and densitometric analyses of the effects of *NC*-siRNA and *KIF3A*-siRNA on expression of SMO, α-SMA, and SRF proteins in MRC-5 cells with or without SiO_2 stimulation. α-Tub was used as a loading control. *P<0.05; **P<0.01. Data are the mean±SD. Statistical analysis was performed using one-way ANOVA and SPSS 20.0.

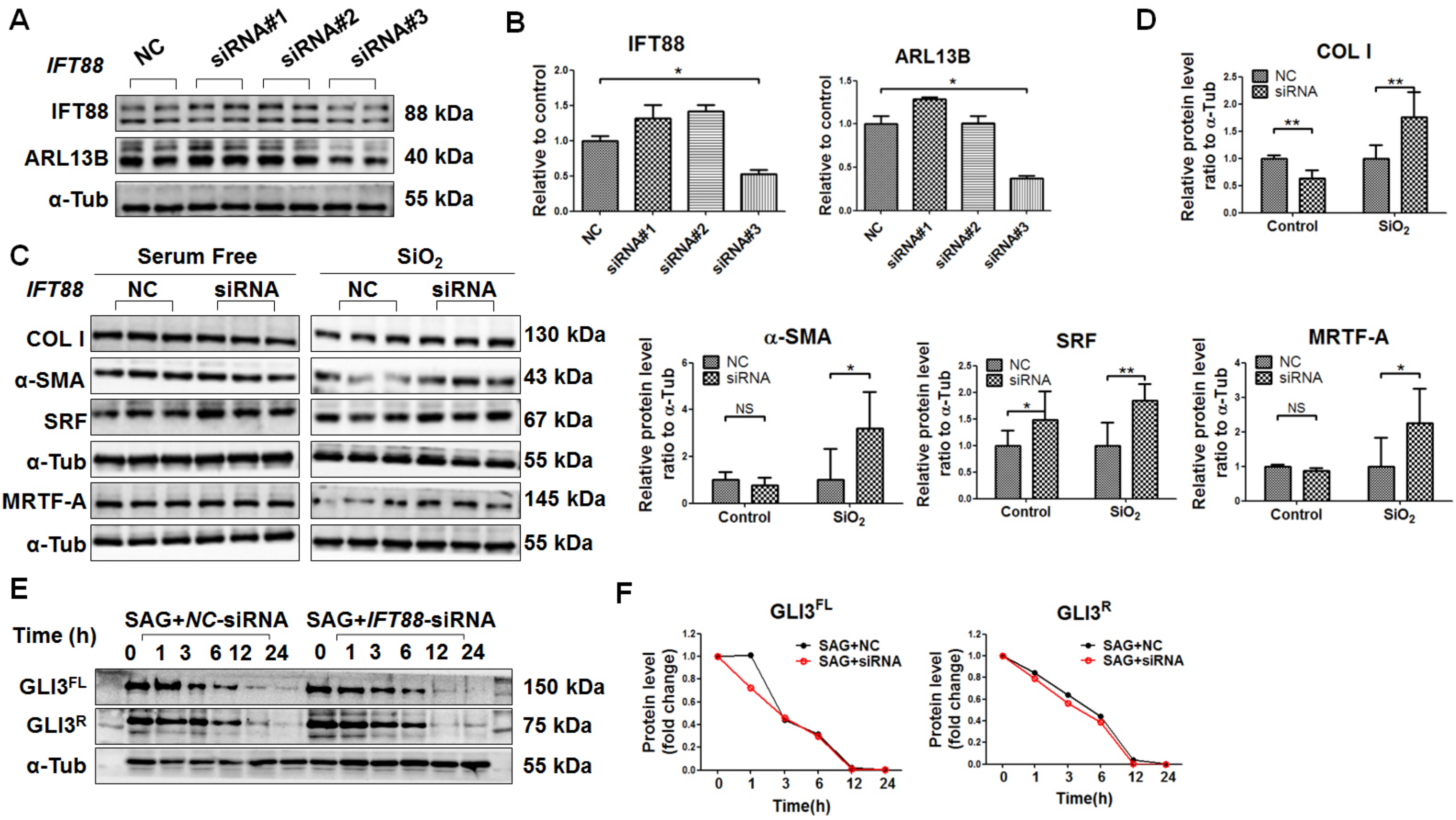

Figure S6 ***IFT88*** **knockdown increases α-SMA-positive myofibroblasts among SiO_2-activated MRC-5 cells.** (A) Western blotting showing the effects of *NC*-siRNA and *IFT88*-siRNA on expression of IFT88 and ARL13B proteins in MRC-5 cells. α-Tub was used as a loading control (n=4). (B) Densitometric analyses of IFT88 and ARL13B protein expression in MRC-5 cells. *P<0.05; **P<0.01. Data are the mean±SD. Statistical analysis was performed using one-way ANOVA and SPSS 20.0. (C, D) Western blotting and densitometric analyses of the effects of *NC*-siRNA and *IFT88*-siRNA on the expression of COL I, α-SMA, SRF, and MRTF-A proteins in MRC-5 cells with or without SiO_2 stimulation. α-Tub was used as a loading control (n=3). *P<0.05; **P<0.01. Data are the mean±SD. Statistical analysis was performed using one-way ANOVA and SPSS 20.0. (E, F) $Gli3^{FL}$ and $Gli3^{R}$ protein levels in MRC-5 cells treated with SAG+*NC*-siRNA or SAG+*IFT88*-siRNA for the indicated periods of time. Levels of $Gli3^{FL}$ and $Gli3^{R}$ protein were assayed by western blotting. α-Tub was used as a loading control. Scatter diagrams are the means of three separate experiments.

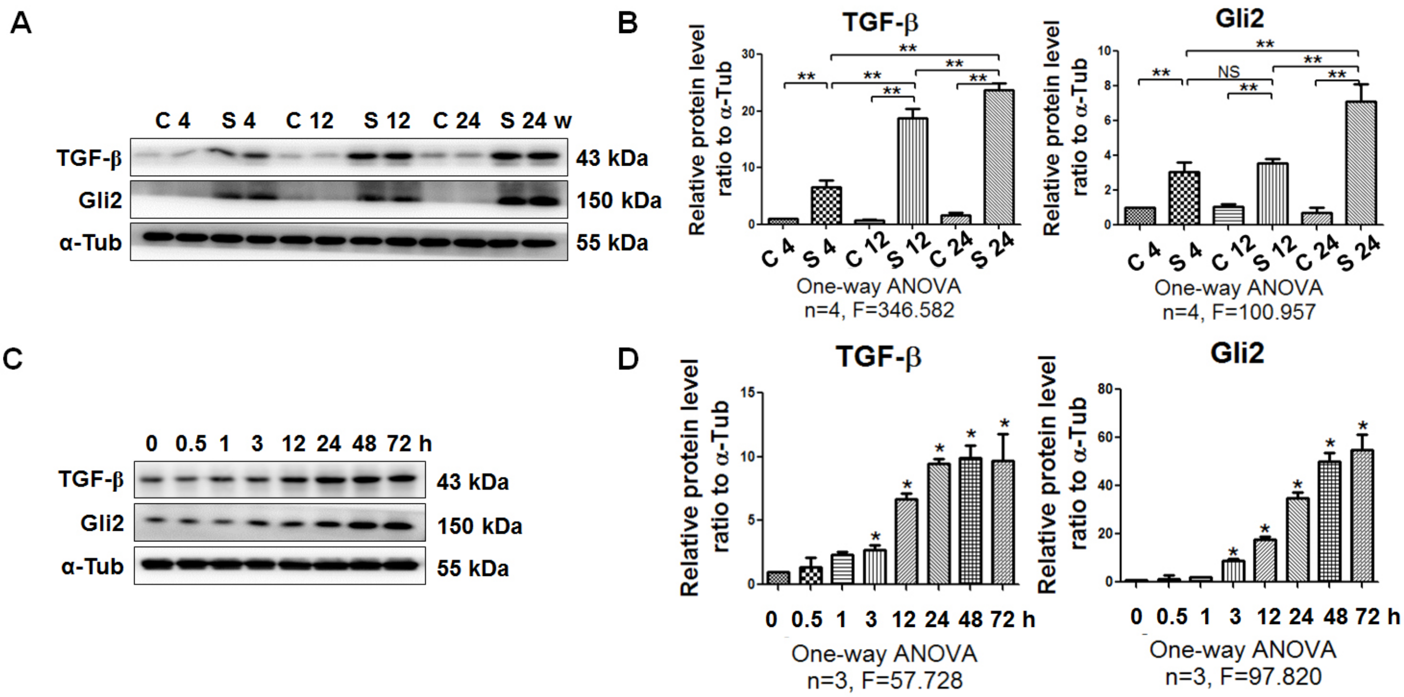

Figure S7 Expression of TGF-β and Gli2 in silicotic rats and SiO_2-stimulated MRC-5. Western blotting demonstrated that expression of TGF-β and Gli2 was increased gradually *in vivo* (Figure 7A, B) and *in vitro* (Figure 7C, D) in a time-dependent manner. Bar graphs are the means±SD. Statistical analysis was performed using one-way ANOVA and SPSS 20.0.

Human (hg38): The binding site of GLI2 located within 1kb upstream of SRF gene in (sample HUMHG02549)

GLI2: binding site[1]	Regulatory range	within upstream 1kb
	Gene reference id	ENSG00000112658.7
	Official gene symbol	*SRF*
	TSS	chr6:43171298
	Gene type	protein_coding
	GLI2 binding locus	chr6:43170333-43171757, Summit: 43171045
	Binding site distance	253
	Motif locus	chr6:43171177-43171187[+], Summit: 43171182
	Motif distance	43171182
	Motif sequence	plus : 5' GGGCCAATGGG 3'<br>minus: 3' CCCGGTTACCC 5'

Human (hg38): The binding site of GLI3 located within 1kb downstream of ACTA2 gene in (sample HUMHG02678)

GLI3: binding site[1]	Regulatory range	within downstream 1kb
	Gene reference id	ENSG00000107796.13
	Official gene symbol	*ACTA2*
	TSS	chr10:88991339
	Gene type	protein_coding
	GLI3 binding locus	chr10:88990122-88992439, Summit: 88991281
	Binding site distance	-59

Figure S8 The binding sites of GLI2 on *SRF* and GLI3 on *ACTA2* (α-SMA). The putative GLI-binding sites (GBS) were identified using an online tool (http://rna.sysu.edu.cn), and the images are the website information.

Supplement Table 1 Demographic features, occupational exposure, and pulmonary function tests of silicosis patients and control subjects

	Stage 0^+ (n=8)	Stage I (n=16)	Stage II (n=16)	Stage III (n=16)	χ^2/F	*P*
Age (mean±SD)	47±7	50±7	45±9	46±7	1.468	0.234
Smoking (n%)						
No	3(37.50)	2(12.50)	6(37.50)	3(18.75)	0.778	0.378
Yes	5(62.50)	14(87.50)	10(62.50)	13(81.25)		
Drinking (n%)						
No	4(50.00)	12(75.00)	12(75.00)	11(68.75)	1.703	0.192
Yes	4(50.00)	4(25.00)	4(25.00)	5(31.25)		
Age at start of work (mean±SD)	29±12	23±5	25±11	24±8	0.987	0.512
Exposure duration (mean±SD)	13±9	21±7	13±8	14±10	2.845	0.048
Pulmonary function						
VC	82.11±11.71	81.83±12.99	81.16±17.21	65.36±15.56*	3.962	0.013
FVC	83.00±15.36	82.41±14.47	81.09±17.71	62.71±18.30*	4.492	0.007
FEV_1	79.81±22.92	76.96±20.20	71.36±19.79	58.01±17.96*	2.863	0.047
FEV_1/FVC (%)	77.73±16.53	75.24±11.61	73.02±15.70	76.51±10.67	0.265	0.850
DL_{CO}	80.39±24.47	80.21±19.86	78.95±17.99	62.71±10.28*	3.087	0.036
RV	119.06±36.32	124.31±32.10	95.79±16.09	87.89±13.38*	6.860	0.001
RV/TLC (%)	40.45±12.72	38.30±12.78	35.52±5.00	37.67±7.17*	2.920	0.047

* P<0.05 vs Stage 0^+ silicosis patients
